# Plant life at the dry limit—Spatial patterns of floristic diversity and composition around the hyperarid core of the Atacama Desert

**DOI:** 10.1371/journal.pone.0233729

**Published:** 2020-05-29

**Authors:** Jonathan Ruhm, Tim Böhnert, Maximilian Weigend, Felix F. Merklinger, Alexandra Stoll, Dietmar Quandt, Federico Luebert

**Affiliations:** 1 Nees Institute for Biodiversity of Plants, University of Bonn, Bonn, Germany; 2 Centro de Estudios Avanzados en Zonas Áridas, CEAZA, La Serena, Chile; 3 Instituto de Investigación Multidisciplinar en Ciencia y Tecnología, Universidad de la Serena, La Serena, Chile; 4 Leibniz Institute of Plant Genetics and Crop Plant Research (IPK), AG ETX, Gatersleben, Germany; 5 Departamento de Silvicultura y Conservación de la Naturaleza, Universidad de Chile, Santiago, Chile; Estacion Experimental de Zonas Aridas, SPAIN

## Abstract

Extreme arid conditions in the Atacama Desert in northern Chile have created a unique vegetation almost entirely restricted to the desert margins along the coast of the Pacific Ocean and the Andean range. In this study we provide data on the desert vegetation along elevational gradients at four localities from the western Andean slopes, between 19° and 21° S. Additionally, zonation of floristic data was explored. Three altitudinal zones could be classified and described in detail for each locality. Conspicuously divergent floras in the Atacama Desert have been recorded in the coastal ‘lomas formations’ and in the Andean desert vegetation, separated by a narrow band of absolute desert. In this study, we investigate the floristic relationships between both regions by implementing similarity analyses for 21 localities from the coastal and Andean deserts in northern Chile. Our results show a drastic east-west divergence in pairwise floristic similarity, which is in stark contrast to a weaker north-south divergence. A biotic barrier, preventing plant exchange from east to west and vice versa, imposed by the hyperarid conditions of the absolute desert, is one possible explanation for this finding. Moreover, the coastal and Andean deserts likely represent ecologically divergent habitats, e.g., in rainfall seasonality. Essential differences in factors determining plant life between both regions have probably contributed to a divergent evolution of the floras. Both explanations–ecological divergence and ecogeographical isolation—are not mutually exclusive, but likely complementary. We also combined floristic data from northern Chile and southern Peru. Similarity analyses of this combined dataset provide first floristic evidence for the existence of a biotic north-south corridor along the western slope of the Andes. Sub-Andean distributions of several species are discussed in the light of floristic connectivity between the Peruvian and Chilean Andean floristic clusters.

## Introduction

Although the Atacama Desert of northern Chile is one of the driest places on earth [1] it harbors a unique and diverse flora, well adapted to this hyperarid environments [2]. With few exceptions (e.g., riverbeds or canyons), vegetation is restricted to the desert margins along the Andean range to the east and at the Pacific coastal range to the west [3].

The climate of the Atacama Desert is characterized by a summer-rainfall zone to the northeast and a winter rainfall zone to the southwest, and a hyperarid zone separating them. In the Andean range north of 24° S, precipitation falls mostly in summer [4]. During austral summer, moisture laden air masses from the Amazonian Basin and the Gran Chaco region pass the Andean range and bring rainfall to the western Andean slopes. Here, annual precipitation increases rapidly with elevation from less than 20 mm at 2,300 m to over 300 mm at 5,000 m [5]. Along the coastal range, precipitation falls mainly in winter. During the austral winter, humid air from the Pacific is transported towards the north Chilean coast due to a northward shift of the Intertropical Convergence Zone and the Pacific anticyclone [6, 7]. However, humid air, originating above the Pacific, is blocked from entering the desert by the topographic barrier of the coastal range and a strong temperature inversion along the western coast of South America [8]. Below the temperature inversion along the coast, moisture is received as fog or drizzle, with thick stratus clouds accumulating and pressed against the slopes of the coastal range, forming a fog zone [9, 10]. The influence of this coastal fog reaches a minimum at the transition between winter-rainfall and summer-rainfall zones. Along the entire length of the Atacama between 19° S and 25° S extreme hyperaridity characterizes the desert [8]. Due to these conditions, the Atacama Desert is characterized by a strong west to east moisture gradient and the desert pampa or absolute desert bears virtually no plant life [11].

Vegetation along the Andean range shows strong altitudinal zonation due to decreasing temperature and increasing available moisture with increasing elevation. Andean desert vegetation, designated as ‘pre-Puna’ in some studies, lies below the high Andean ‘tolares’ or Puna vegetation at elevations of typically 3,150 to 3,850 m [12]. Andean desert vegetation has been poorly investigated so far, whereas high Andean vegetation is quite well documented [12–16]. Most plants in the Andean desert only emerge in those rare years with summer rainfall reaching lower elevations. At the coastal range, however, vegetation has been studied extensively [17]. The coastal flora is largely endemic to the coastal ‘lomas formations’ and arguably distinct from that of the neighboring Andes [18]. This, however, has not been evaluated with floristic data. Likewise, there is essentially anecdotal evidence for levels of endemism along the Andes.

Several studies postulated the existence of a phytogeographical barrier separating the Peruvian from the Chilean coastal desert on the basis of floristic inventories and similarity analyses [18–22]. Floristic connections between Andean deserts of Chile and Peru were first suggested by Schwarzer et al. [23]. Furthermore, based on floristic and faunistic data, Moreno et al. [24] postulated a north-south corridor facilitating biotic exchanges along the Andes. Phylogenetic studies of several plant groups [25–27] tend to support this idea. An isolated evolution of the coastal and the Andean deserts floras, respectively, has already been proposed by Rundel et al. [18]. However, we are not aware of any study evaluating either the isolation of the coastal versus Andean floras or the floristic connections between the Chilean and Peruvian Andean deserts. In the present study, we provide novel floristic data sets for the Andean desert vegetation and altitudinal transects in northern Chile between 19° S and 21° S.

Our aims are to a) give the first description of the desert vegetation along four altitudinal transects in northern Chile and b) explore the phytogeographic relationships between the coastal and Andean deserts of northern Chile and southern Peru using cluster analyses based on floristic data. We base our study on original data here presented, and previously published floristic data from Andean sites in northern Chile and southern Peru as well as from a range of localities along the southern Peruvian and northern Chilean coast.

## Material and methods

### Study areas

**Fig 1:**
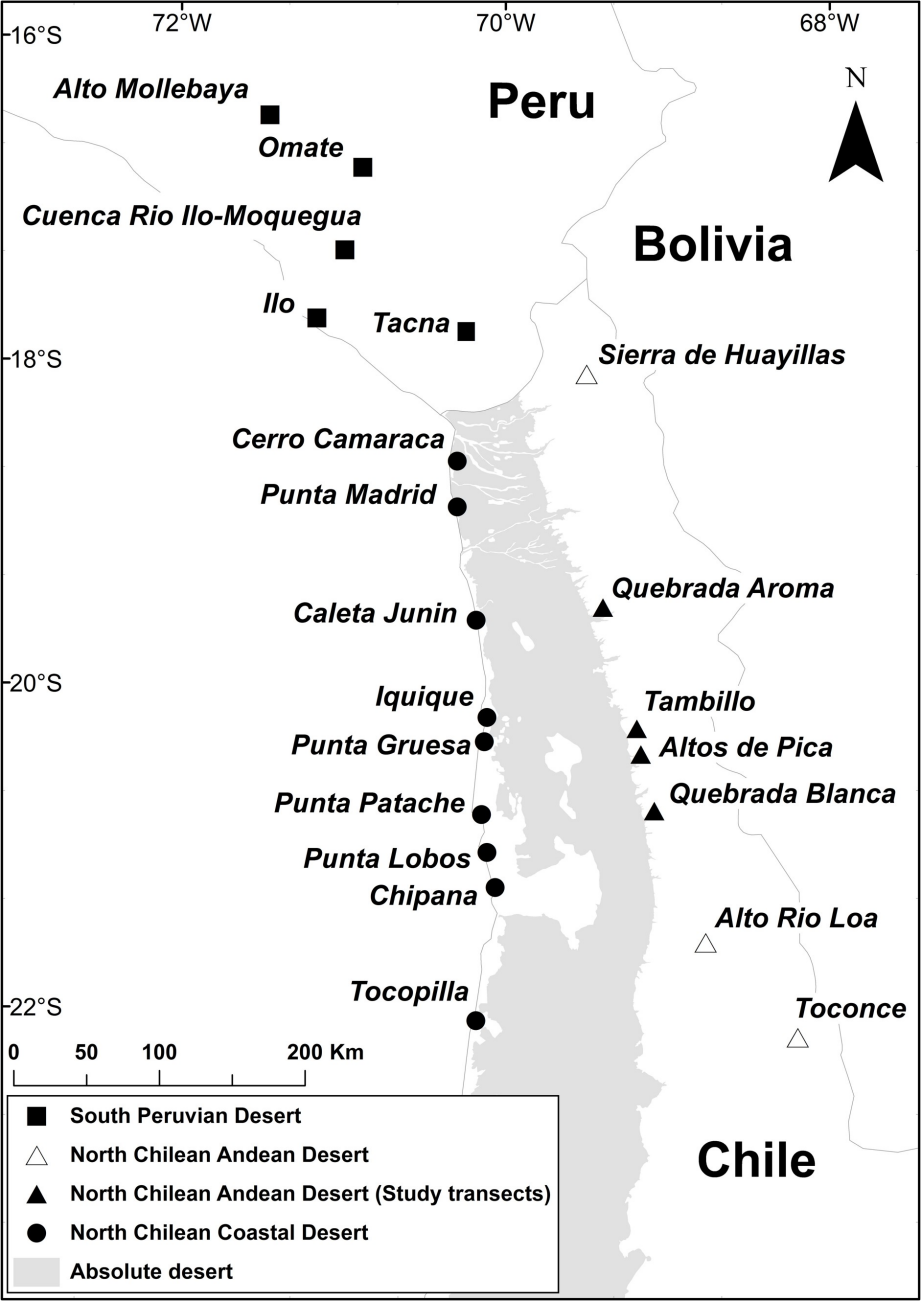
Overview of localities in southern Peru and northern Chile. Area in gray indicates the absolute desert according to Luebert and Pliscoff [2]. Map created using ArcMap 10.6^™^ (version 10.6.0.8321) included in ArcGIS Desktop 10.6 ^®^ (version: 10.6.0.8321) software by ESRI. Country boundaries were provided by ESRI [28]. Information on the extent of the absolute desert from Luebert and Pliscoff [2], accessed as shape file at the Zenodo online repository (doi: 10.5281/zenodo.60800).

In March 2017, floristic surveys were carried out at four localities along the Andean desert of the Tarapacá region in northern Chile (Fig 1). Climate data (Table 1) indicate remarkably humid weather conditions for this area in 2017 in comparison to other years. The following transects were studied:

1. Quebrada Aroma (19.52875° S, 69.37502° W to 19.57025° S, 69.46659° W): Vegetation of the Andean slopes along Quebrada Aroma was assessed between 2,600 and 2,200 m with five plots. Due to accessibility, this transect did not reach the transition towards the Andean vegetation. Below 2,400 m, vegetation is restricted to the cooler and wetter climate of the quebradas. The vegetation around Quebrada Aroma is dominated by shrubby species such as *Aphyllocladus denticulatus* (J. Remy) Cabrera (Asteraceae) and *Atriplex glaucescens* Phil. (Amaranthaceae) as well as columnar cacti such as *Browningia candelaris* (Meyen) Britton & Rose and smaller clustering species such as *Cumulopuntia sphaerica* (C.F. Först.) E.F. Anderson (Cactaceae).
2. Altos de Pica (20.38928° S, 69.08904° W to 20.48502° S, 69.22031° W): This transect is located at the slopes above the town of Pica along the road Pica—Salar de Huasco. Vegetation was assessed in ten plots between 3,200 and 2,200 m. Vegetation was only found above 2,500 m. Characteristic plants are shrubs and subshrubs such as *Atriplex glaucescens*, *Tiquilia paronychioides* (Phil.) A.T. Richardson (Ehretiaceae), but also forbs, for example the geophytic *Mastigostyla cyrtophylla* I.M. Johnst. (Iridaceae).
3. Quebrada Blanca (20.80247° S, 69.02584° W to 20.82217° S, 69.14174° W): Eleven plots were investigated between 3,200 to 2,100 m along the road connecting the copper mine of Quebrada Blanca with the Pan-American Highway. The absolute desert starts below 2,100 m. Besides the shrub *Atriplex glaucescens*, several herbaceous species were dominant, such as *Tetragonia microcarpa* Phil. (Aizoaceae), *Pectocarya anomala* I.M. Johnst. (Boraginaceae) and *Chorizanthe commissuralis* J. Remy (Polygonaceae).
4. Tambillo (20.30425° S, 69.11324° W to 20.26914° S, 69.32291° W): Starting at 3,200 m, the altitudinal gradient follows the road between Salar de Huasco and the Pan-American Highway down to 2,000 m. In this elevation range, vegetation was assessed in twelve plots. From 2,300 m downwards, no plant species were recorded. Typical species in this area are the subshrub *Tiquilia grandiflora* (Phil.) A.T. Richardson (Ehretiaceae) along with *Munroa andina* Phil. (Poaceae) and *Mirabilis trollii* Heimerl (Nyctaginaceae).

**Table 1:** Annual precipitation (mm) in three localities around the study area recorded from 2010 to 2018.

Year	Sibaya (2830 m)	Poroma (2880 m)	Guataconco (2460 m)
	(19.53722° S, 69.20500° W)	(19.87166° S, 69.18222° W)	(20.92750° S, 69.05277° W)
2010	6.8	22	21.5
2011	35.5	157	71.3
2012	82.3	154	53.1
2013	49.9	56	3
2014	23.6	33	0
2015	49.3	80.5	20
2016	33.4	55	0
**2017**	**154.4**	**110**	**36**
2018	27.6	53	7.4
Ø	51.42	80.6	23.59

Data was obtained from the Dirección General de Aguas, Chile (DGA) (*http*:*//dga*.*cl*, accessed: 11/05/2019).

### Vegetation data and plant identification

The actual desert vegetation was segregated from the Andean vegetation by the absence of species typical for the high Andean tolares (e.g., *Fabiana ramulosa* (Wedd.) Hunz. & Barboza; [2]). Starting at the lower edge of the tolares flora, floristic inventories were conducted in plots of 0.1 ha (32 × 32 m) along altitudinal transects with one plot randomly located every 100 m elevation. Sampling was stopped after two consecutive plots with no plants as evidence for having reached the absolute desert. For each plot, all plant species were recorded, as well as general plant cover and the cover-abundance of each species according to Braun-Blanquet [29, 30]. For every species record, a herbarium specimen was collected and a preliminary name was assigned to each specimen. Nearby species (outside the plots) were also recorded, to enrich the information of floristic diversity for each location following Mueller-Dombois and Ellenberg [31] (p. 61). Specimens were deposited in the herbaria of the University of Bonn, Germany (BONN) and the University of La Serena, Chile (ULS; S1 Table). Supplementary tables from this study are available from the CRC database [32]. As of March 2020, Chile has not regulated plant collecting according to the Convention of Biological Diversity (CBD), so no collecting permit was required. Field studies did not involve endangered or protected species.

Determination of herbarium specimens was carried out at Bonn University. Taxonomic studies on the genera and species within the focus region were used for specimen identification (S2 Table). If these were not available, original descriptions of species were studied and, whenever necessary, high-resolution scans of type specimens were used for comparison (https://plants.jstor.org/). Scientific names were assigned and synonyms revised according to Zuloaga et al. [33]. Where species determination was not possible, specimens were assigned to morphospecies or labeled for pending confirmation. Information on lifeforms was taken from Zuloaga et al. [33] and adjusted to the main groups of plant lifeforms according to Ellenberg and Mueller-Dombois [34]. Missing information was complemented either by consulting species descriptions or by an inspection of conspecific herbarium specimens.

For a larger scale floristic comparison, additional data on the Andean desert flora of northern Chile was compiled from Villagrán et al. [12, 13] and Teillier [14]. Floristic information on localities from the coastal desert in northern Chile as well as the coastal and Andean deserts in southern Peru were taken from Pinto and Luebert [21], Schwarzer et al. [23] and Arakaki et al. [35]. Scientific names of species were assigned and revised according to Brako and Zarucchi [36] for Peru and Zuloaga et al. [33] for Chile. A complete list of species across all these localities is given in S3 Table. See Fig 1 for all localities included in the analyses.

### Data processing

#### Coverage and composition of the transects

For the vegetation analysis along the elevational gradients of the studied transects (Quebrada Aroma, Altos de Pica, Quebrada Blanca and Tambillo), data were visualized and inspected in R 3.4.2 [37] and RStudio 1.1.383 [38]. Estimates of cover-abundance [30, 31] for species from the studied transects were transformed to average cover values (in %) according to Tüxen and Ellenberg [39, 40]. S4 Table contains a comprehensive species list, average cover values of all species from the transects, their life form as well as general information about plots. Lifeform spectra based on vegetation cover and species numbers were generated for the five main groups of self-supporting vascular plants for each elevational plot within the studied transects [34]. For lifeform spectra based on vegetation cover the percentage of each lifeform was calculated from the sum of all average cover values of species per plot. The four to five most dominating species were identified for each plot based on average cover values. Elevational abundance profiles were generated for all dominant species across the whole range of transects. If a species was not recorded for an individual plot, but found in plots below and above along a studied transects, the missing data were imputed with the average of the upper and lower plot cover values of that species.

#### Similarity analyses

Similarity analyses and a classification of floristic data were performed using the R-package vegan 2.5–6 to explore floristic zonation along altitudinal gradients [41]. Pairwise floristic distances between plots were calculated using the Sørensen Similarity Index [42]. Similarity analyses were based on presence/absence data of species. Species recorded outside the plots were excluded from these analyses. Hierarchical agglomerative clustering (HAC), using Ward’s minimum variance criterion [43], was employed for the classification of plots. Ward’s method was selected on the basis of silhouette plots [44] among four clustering methods (average linkage, complete linkage, single linkage, ‘Ward’s method’), using the R-package fpc 2.2–3 [45].

#### Floristic comparison

Floristic relationships between localities from coastal and Andean deserts of northern Chile and southern Peru were examined by conducting similarity analyses and classification of localities using the same parameters as described above. Regarding the floristic data, each transect was treated as a sample, pooling the species lists from all plots in the transect. In addition, the species records from outside the plots were included. Similarity analyses were then performed based on presence/absence data of species for each locality. A dendrogram and a heatmap were created with the R-packages gplots v3.0.1.1 [46] and factoextra 1.0.5 [47] to display the results of the similarity analysis. In addition, an ordination analysis based on floristic dissimilarities by Principal Coordinates Analysis (PCoA) [48] was conducted using the R-package ade4 1.7.13 [49]. Results of classification and ordination were subsequently combined and groups were visualized along the first two PCoA axes. Geographical distances were plotted against floristic dissimilarities. To calculate geographical distances between localities, the R-package geosphere 1.5.10 [50] was employed.

## Results

### Flora of the studied transects

The 153 herbarium specimens collected along the four studied transects were assigned to 81 species and morphospecies in 62 genera and 26 families (S1 Table). The most speciose families encountered were Asteraceae (12 spp.), Solanaceae (8 spp.) and Poaceae (7 spp.). Removing species recorded outside the plots reduced the species total to 68, including morphospecies.

### Physiognomy and dominant species of the studied transects

#### Quebrada Aroma

**Fig 2:**
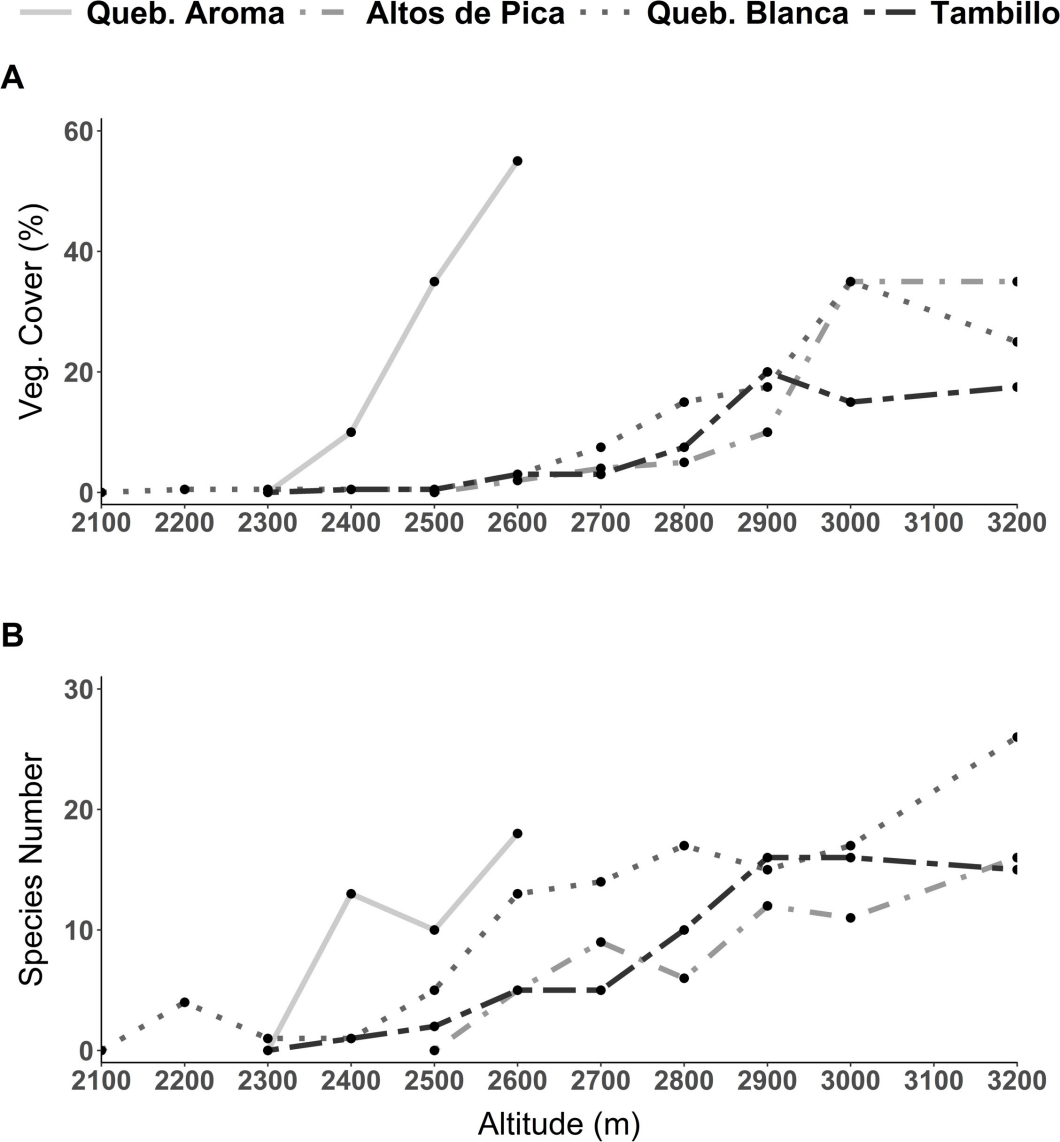
Vegetation changes across the elevation gradient from the four studied transects. **A)** Vegetation cover in percentage. **B)** Species richness in total numbers.

**Fig 3:**
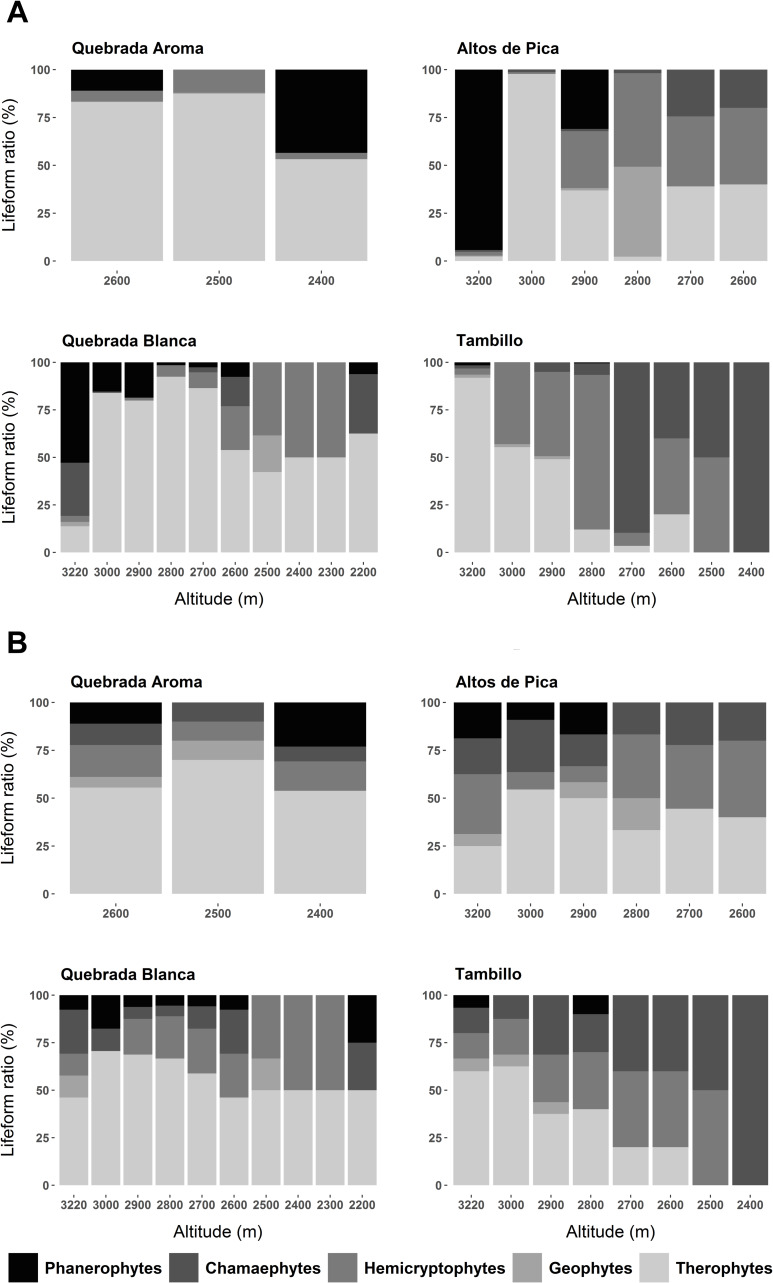
Lifeform spectra of the five dominant species for each elevational plot in the four studied transects. **A)** Lifeform spectra based on vegetation cover. **B)** Lifeform spectra based on species number.

**Fig 4:**
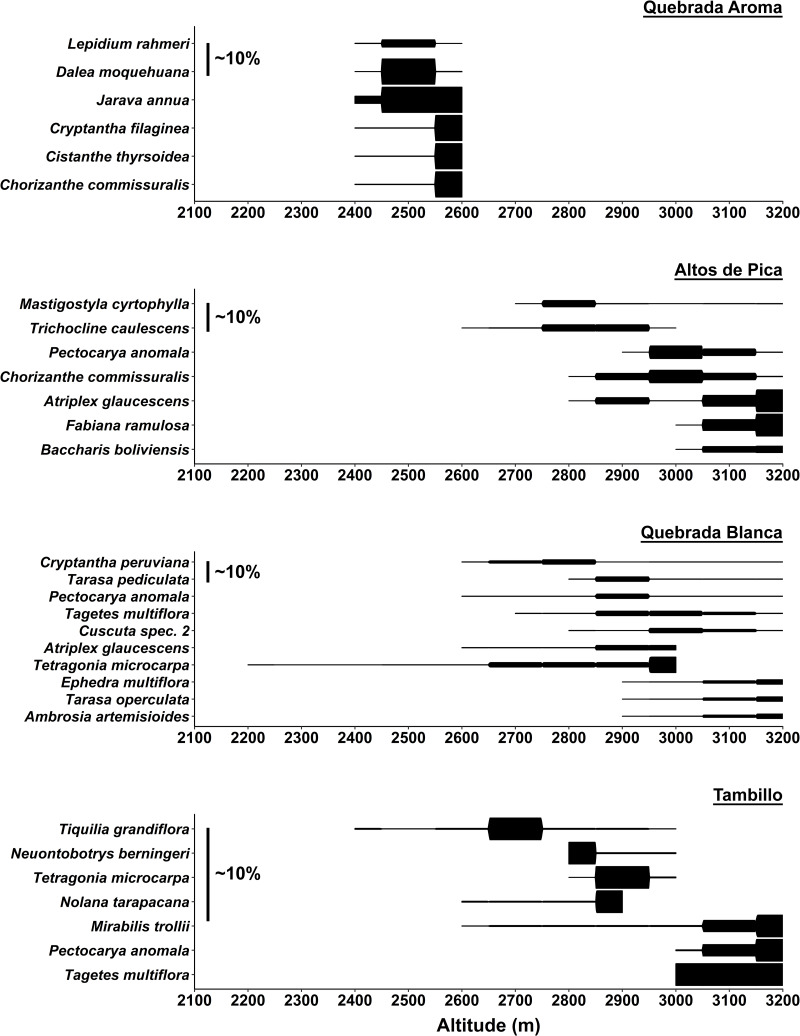
Abundance profiles of most dominant species along the four studied transects. Abundance profiles are based on average cover values given in percentage (%) according to Tüxen and Ellenberg [39, 40]. Most dominant species were selected by highest cover values. For each transect scalebars indicate average plant cover of about 10%.

Species number as well as vegetation cover showed a stronger decrease at lower elevations compared to the other studied transects (Fig 2). As indicated in Fig 2A vegetation cover in this transect is the highest of the whole study area at each elevation. Across the whole range of the elevation gradient, hemicryptophytes and therophytes dominated the vegetation in both species number and abundance (Fig 3). Regarding vegetation cover woody species increased close to the desert center (Fig 3A). *Jarava annua* (Mez) Peñailillo was consistently encountered across all elevations in high numbers (Fig 4). At 2,600 m, besides *Jarava annua*, the most abundant species were *Chorizanthe commissuralis* J. Rémy., *Cistanthe thyrsoidea* (Reiche) Peralta & D.I. Ford, *Cryptantha filaginea* (Phil.) Reiche as well as *Atriplex glaucescens* (Fig 4). *Dalea moquehuana* J.F. Macbr. and *Lepidium rahmeri* dominated at 2,500 m (Fig 4). At 2,400 m, vegetation was dominated by *Jarava annua* and *Atriplex glaucescens*.

#### Altos de Pica

A sharp decline in vegetation cover by approximately two-thirds (Fig 2A) appeared from 3,000 to 2,900 m. Species number was reduced more gradually toward the lowest point close to the absolute desert (Fig 2B). At 3,200 m, phanerophytes dominated in vegetation cover but became considerably less prominent at lower elevations (Fig 3A). Additionally, an overall shift from predominantly woody to predominantly herbaceous species from higher to lower elevations was observed (Fig 3). At 3,200 m the woody species *Baccharis boliviensis* (Wedd.) Cabrera, *Atriplex glaucescens* and *Fabiana ramulosa* were the most abundant species (Fig 4) marking the transition to the high Andean flora. At 3,000 m the herbaceous species *Chorizanthe commissuralis* J. Rémy and *Pectocarya anomala* were common. At 2,900 and 2,800 m, the most abundant species were *Pectocarya anomala*, *Atriplex glaucescens*, *Trichocline caulescens* Phil., *Mastigostyla cyrtophylla* and *Chorizanthe commissuralis*. Below 2,800 m, dominant species could not be distinguished.

#### Quebrada Blanca

Between 3,000–2,900 m vegetation cover decreased by about half (Fig 2A). Regarding species number, there were marked changes between 3,200 and 3,000 m, as well as between 2,600 and 2,400 m. In each case species number decreased by about a third (Fig 2B). Near the transition towards the Andean ‘tolares’ vegetation (3,200 m), phanerophytes and chamaephytes dominated the vegetation (*Ambrosia artemisioides Meyen* & Walp. ex Meyen, *Ephedra multiflora* Phil. ex Stapf, *Atriplex glaucescens*, *Tarasa operculata* (Cav.) Krapov.) (Figs 3 and 4). At 3,000 m, therophytes became the dominant lifeform. The most common species were *Pectocarya anomala*, *Tarasa pediculata*, *Cryptantha peruviana* I. M. Johnst., *Tetragonia microcarpa* and *Tagetes multiflora*. Intermediate and low elevational zones were not characterized by dominant species. From 2,400 to 2,200 m, *Tetragonia microcarpa* and *Mirabilis trollii* were the only recorded species found in several plots. Towards the absolute desert, the woody species *Atriplex imbricata* (Moq.) D. Dietr. was found again along with the therophytes, albeit with low abundance (S4 Table).

#### Tambillo

From 2,900 to 2,700 m vegetation changed significantly regarding plant cover and species number, which were both reduced by more than half (Fig 2A and 2B). Vegetation cover was in general relatively low in comparison to the other transects (Fig 2A). At 3,200 m, hemicryptophytes and therophytes represented the prevailing lifeforms (Fig 3). Even though woody species tended to increase downhill with increasing aridity, hemicryptophytes and therophytes continued to dominate the vegetation from 3,000 to 2,600 m. At 2,400 m only Chamaepyhtes were recognized. Between 3,200–3,000 m *Tagetes multiflora*, *Pectocarya anomala* and *Mirabilis trollii* dominated (Fig 4). *Nolana tarapacana* (Phil.) I.M. Johnst., *Tetragonia microcarpa*, *Neuontobotrys berningeri* O.E. Schulz and *Tagetes multiflora* dominated at lower elevations between 2,900–2,800 m. *Tiquilia grandiflora* became prevailing at 2,700 m. At 2,500 m and 2,400 m only single individuals of *Tiquilia grandiflora* and *Mirabilis trollii* were found.

### Altitudinal vegetation zones

**Fig 5:**
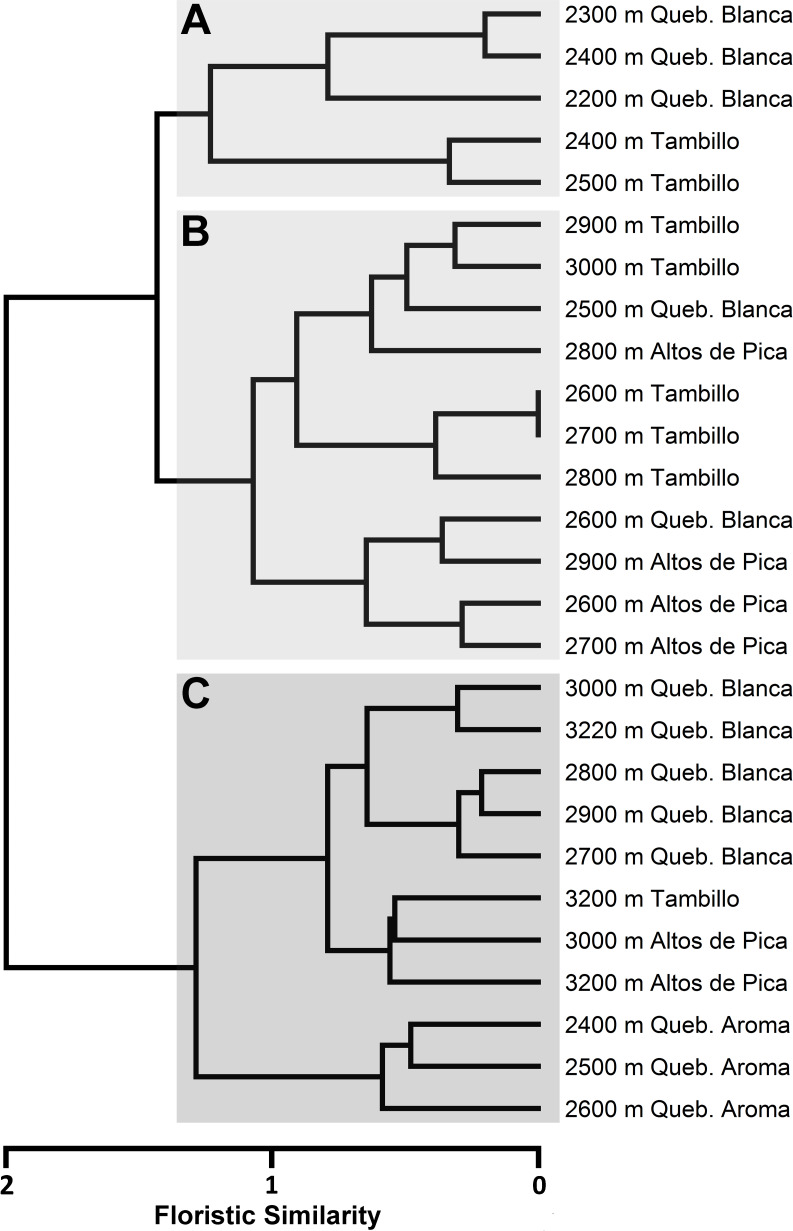
Classification of floristic distance between elevational plots from the four studied transects. Clustering was conducted with the Ward’s minimum variance method. Similarity analysis was performed using Sørensen index**. A)** Cluster of plots from lowest elevations. **B)** Cluster of plots from intermediate elevations. **C)** Cluster of plots from high elevations.

**Fig 6:**
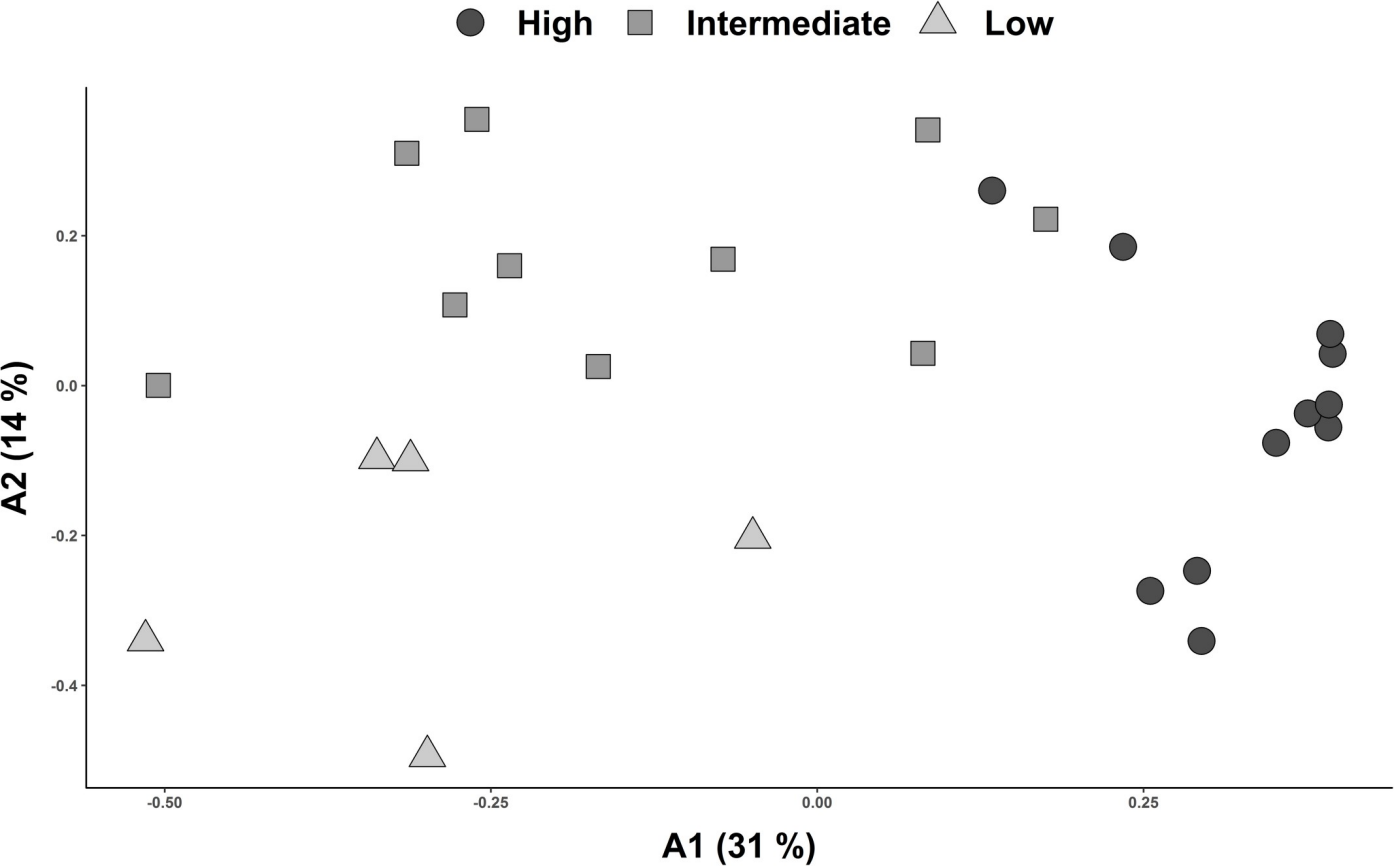
Ordination analysis of pairwise floristic similarity (Sørensen index) including plots from all four studied transects. Plots are assigned to altitudinal vegetation zones (A, B, C) retrieved from cluster analysis (Fig 4). X-and y-axis correspond to the first two axes resulting from Principal Coordinates Analysis (PCoA).

Classification of floristic similarity between the study plots revealed an elevation related clustering into three major altitudinal zones of desert vegetation along the Andes. These clusters were designated as Low (A), Intermediate (B) and High (C) (Fig 5; Table 2). Ordination analysis of the study plots assigned to the previously designated altitudinal zones and visualized along the first two axis (A1 and A2) of PCoA further endorse the results of the classification analysis (Fig 6). Across all transects, the vegetation zones identified were characterized by specific species found across localities. Most constantly occurring species of the high vegetation zones (C) were *Lepidium rahmeri* Phil., *Tagetes multiflora* Kunth, *Mastigostyla cyrtophylla*, *Tetragonia microcarpa* and *Atriplex glaucescens* (Table 2 and S4 Table). In the intermediate vegetation zone (B), *Tetragonia microcarpa*, *Mirabilis trollii*, *Mastigostyla cyrtophylla* as well as *Munroa andina* were consistently encountered. In the Low vegetation zones (A), *Mirabilis trollii* was the only species found in most plots (Table 2 and S4 Table). Elevational abundance profiles of dominant species for each transect are provided in Fig 4.

**Table 2:** Elevational ranges (m) of vegetation zones for each studied transect.

	Low		Intermediate		High	
	min	max	min	max	min	max
**Quebrada Aroma**	-	-	-	-	2400	2600
**Altos de Pica**	-	-	2600	2900	3000	3200
**Quebrada Blanca**	2300	2400	2500	2600	2700	3220
**Tambillo**	2400	2500	2600	3000	-	3200

### Floristic relationships with other localities in Chile and Peru

**Fig 7:**
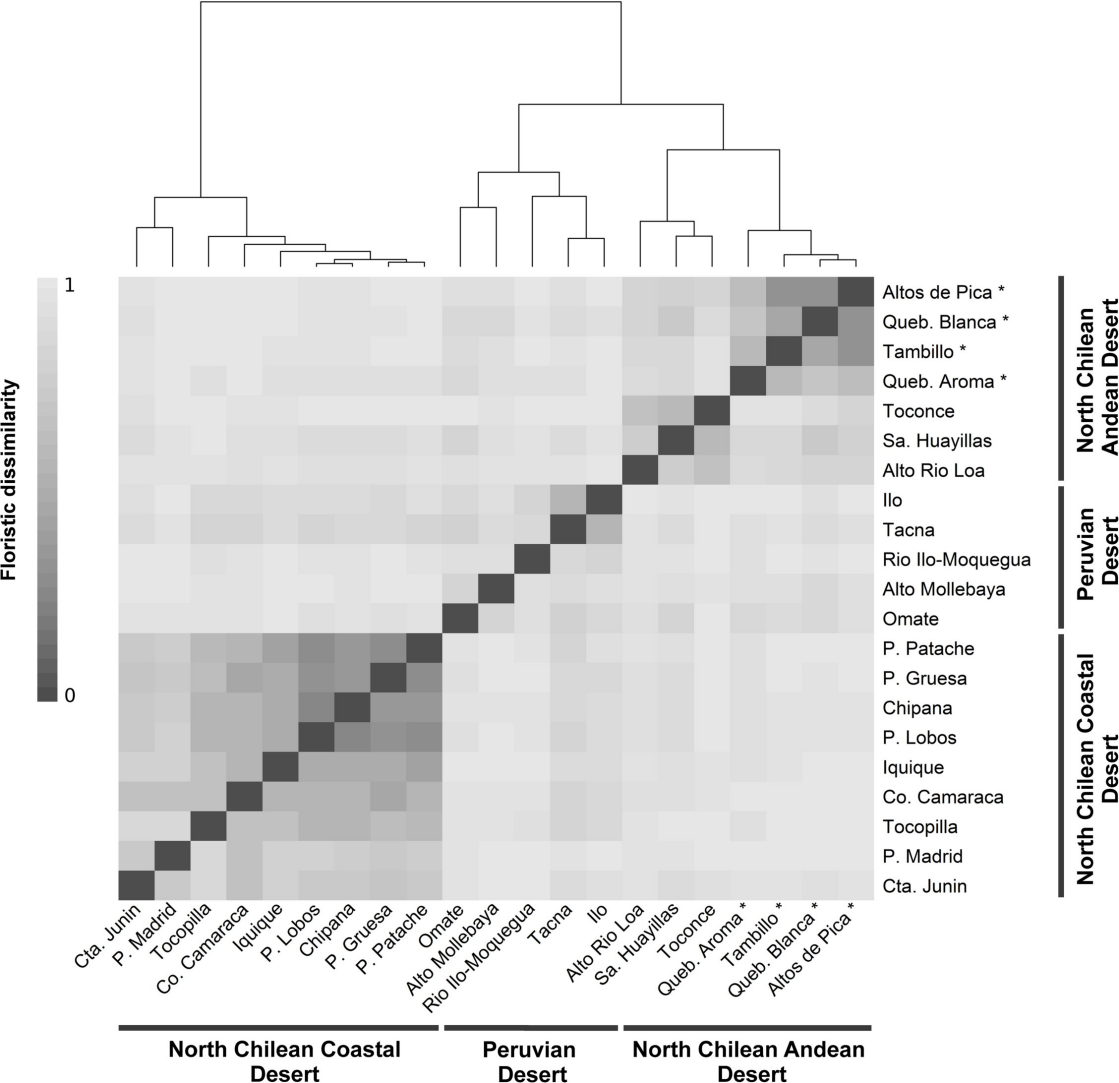
Heatmap of localities from the three floristic clusters derived from the dendrogram on the top (North Chilean Coastal Desert, North Chilean Andean Desert and Peruvian Desert). Classification was conditioned by Ward’s minimum variance method. Similarity analysis was performed using Sørensen index. ‘*’ indicate localities of the elevational gradients presented in this study.

Classification of floristic pairwise distances yielded three distinct clusters: (1) North Chilean Coastal Desert (Cta. Junin, P. Madrid, Co. Camaraca, Iquique, P. Lobos, Chipana, P. Gruesa, P. Patache), (2) North Chilean Andean Desert (Alto Rio Loa, Sa. Huayillas, Toconce, Queb. Aroma, Queb. Blanca, Altos de Pica, Tambillo) and (3) Peruvian Desert (Omate, Alto Mollebaya, Rio Ilo-Moquegua, Tacna, Ilo) (Figs 1 and 7). The North Chilean Coastal Desert cluster was retrieved separately whereas the Peruvian Desert and North Chilean Andean Desert clusters were placed together. Two subgroups could be identified within the North Chilean Andean Desert cluster: One comprising the four localities from the Tarapacá region of this study, the other combining localities from regions further north (Arica y Parinacota) and south (Antofagasta), compiled from the literature. Within the Peruvian Desert cluster, three localities (Rio Ilo-Moquegua, Tacna, Ilo) were separated from the Andean sites Omate and Alto Mollebaya. In the North Chilean Coastal Desert cluster, a core-group of very similar localities south of Iquique was retrieved together with increasingly dissimilar localities towards the north.

**Fig 8:**
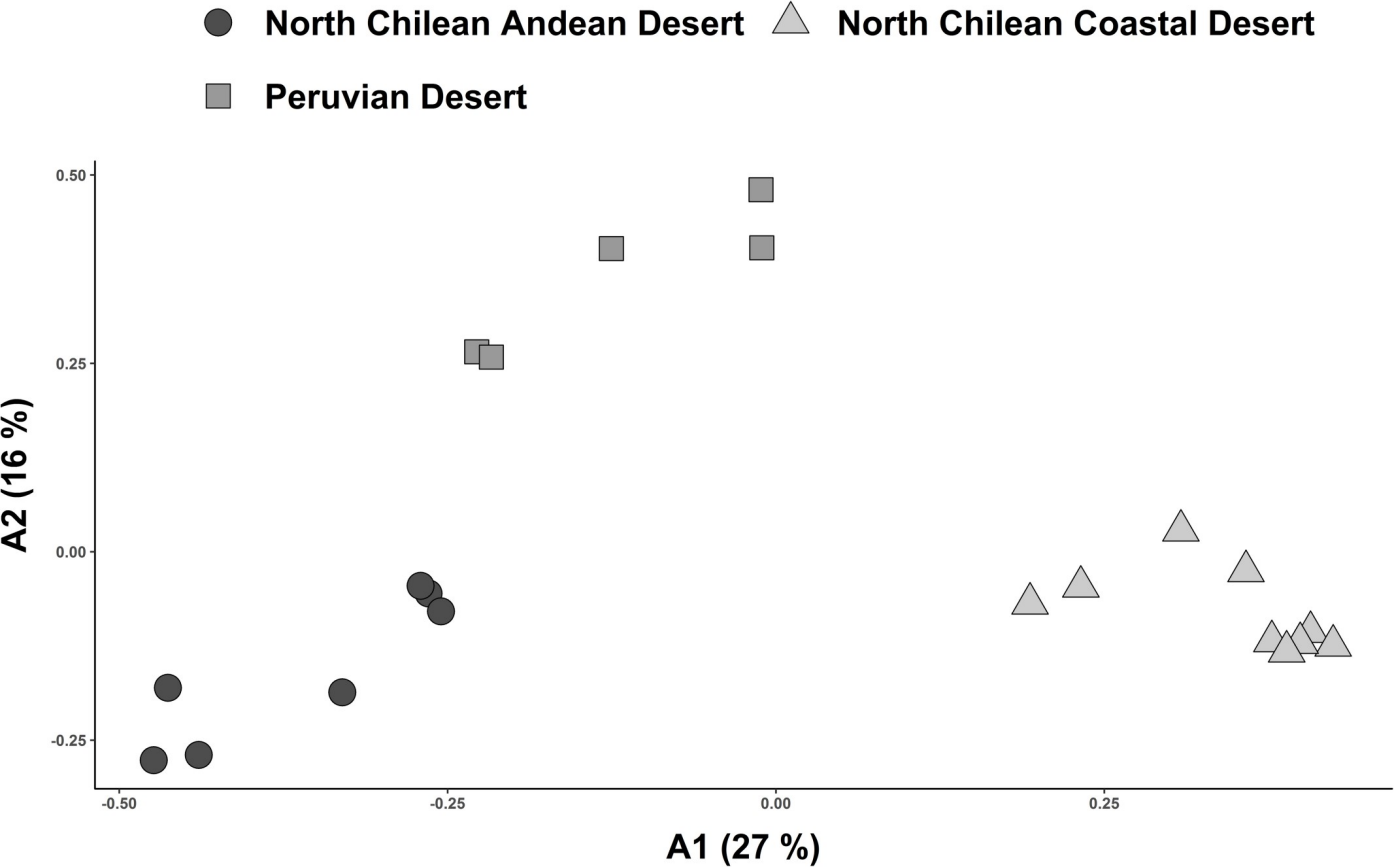
Ordination of values for pairwise floristic similarity (Sørensen index) including Chilean and Peruvian localities from the coast and Andes. Plots are assigned to altitudinal vegetation zones retrieved from cluster analysis (Fig 7). X-and y-axis correspond to the first two axes resulting from Principal Coordinates Analysis (PCoA). The three floristic clusters are placed as clearly separated groups.

**Fig 9:**
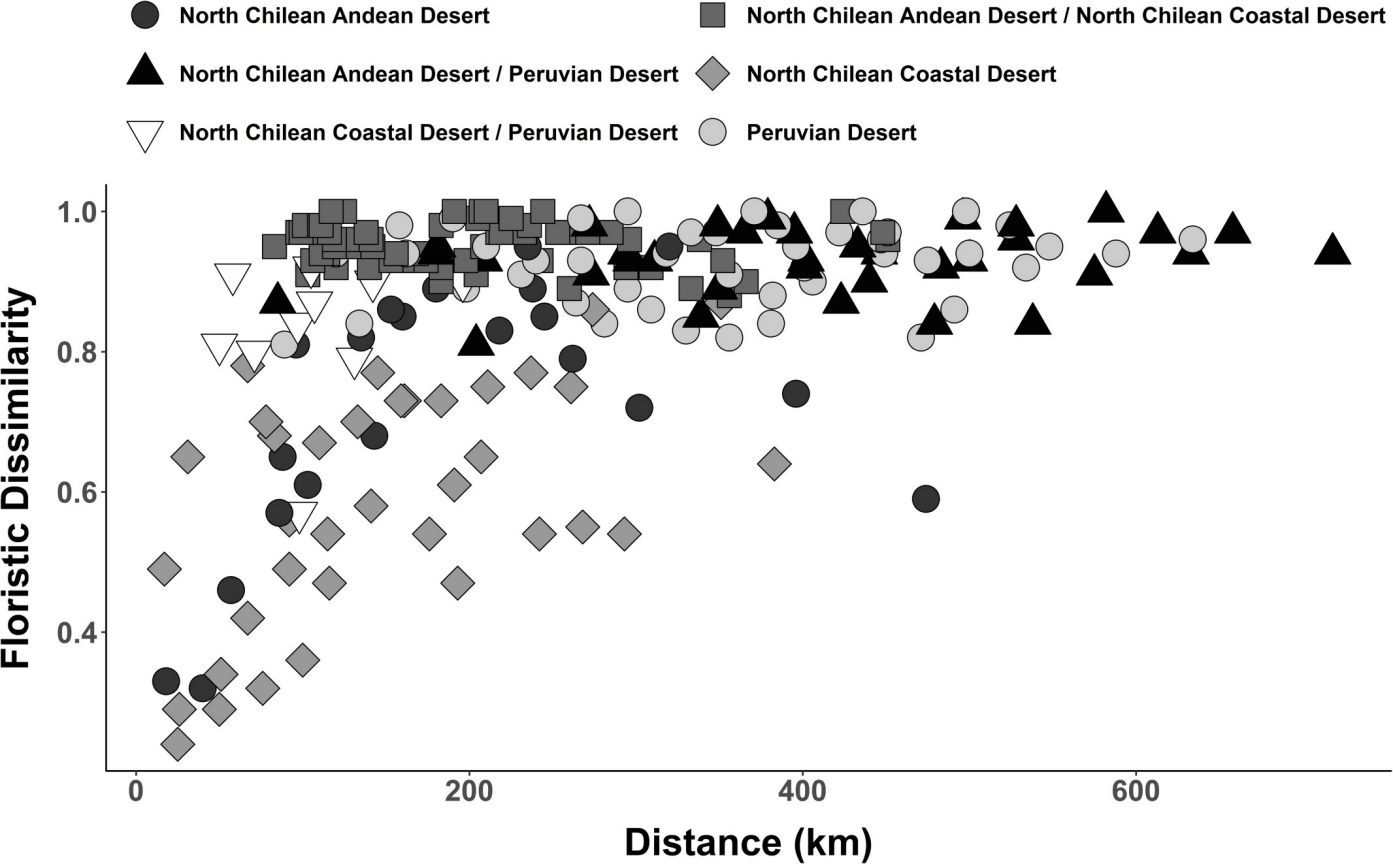
Floristic dissimilarity in relation to geographical distance shown for localities within each floristic cluster in comparison to localities from different floristic clusters.

Coincident with the results of the classification, ordination placed the three floristic clusters as clearly separated groups (Fig 8). Separation along the axis with the most explanatory power (A1: 27%) further underscored the predicted affinity between the Peruvian Desert cluster and North Chilean Andean Desert cluster as separated from the North Chilean Coastal Desert cluster. Pairwise floristic dissimilarities, compared to geographical distance between North Chilean Andean Desert and coastal localities, were nearly always higher than those within the individual clusters (Fig 9). There was a trend to increasing floristic dissimilarity within each floristic cluster with geographical distance along a north-south gradient. Floristic dissimilarity between the North Chilean Andean Desert and Peruvian Desert was high (> 0.8), regardless of geographical distance. The same was true for the comparisons between the North Chilean Coastal Desert and the Peruvian Desert. From a total of 281 species recorded in the North Chilean Coastal Desert and North Chilean Andean Desert cluster, only 14 species were common to both regions (S5 Table), amounting to approximately 5%. The Peruvian Desert and North Chilean Andean Desert cluster shared about 7% of the species (39 of 544) (S6 Table).

## Discussion

### Altitudinal vegetation zonation of desert flora along studied transects

This is the first study proposing an altitudinal vegetation zonation for the Andean Atacama Desert. Hierarchical agglomerative clustering as well as ordination (PCoA) show concordant results, underscoring that zonation is found independently of specific analytical tools.

Our results are based on data gathered in one year of field work. Díaz et al. [51] pointed out that it takes at least six years of observations to record over 90% of all species present in a given area in the Atacama Desert, and that precipitation is the main driver for interannual changes in productivity and plant species richness. We conducted floristic sampling along the studied transects in a rather rainy year (Table 1), probably leading to enhanced plant proliferation. Hence, the vegetation and floristic data of our study also require completion with data collected across a longer period of time. However, floristic data for the Andean desert in northern Chile is still rather sparse and discontinuous and literature often only provides outdated floristic inventories with imprecise geographical context or sampling methods (e.g., Saiz et al. [52]). This hampers robust analyses of latitudinal relationships of floristic composition along the Andean desert. In order to explore latitudinal changes in the zonation of the Andean desert vegetation in northern Chile, complementary and consistent floristic inventories of this region are highly desirable.

Villagrán et al. [12] reported a reduction in the amplitude of desert or ‘pre-Puna’ vegetation from north to south along the Chilean Andes. This correlates with a gradual decrease of summer precipitation [8]. As a result, the absolute desert reaches higher elevations and approaches the Andean tolares more closely at around 25° S [11, 15, 53]. Arroyo et al. [11] report that along the western slopes of the Andes, between 18° S and 19° S, woody species increase, whereas annuals decrease in number with increasing aridity towards the absolute desert. They suggest that drought-resistant woody perennials are better adapted to the extreme desert environment than annuals. Based on our data, this pattern can only be confirmed in one transect in (‘Tambillo’). Although slightly less frequent, therophytes and/or hemicryptophytes are still highly represented even in plots at lower elevations (Fig 3). The conclusion of Arroyo et al. [11] thus does no hold true across the entire region. This may be due to the fact that our sampling was conducted during a rainy year, thus capturing the emergence of these lifeforms. Unfortunately, Arroyo et al. [11] provide no information on climatic conditions during their plant sampling. Díaz et al. [51] suggest that seed banks of annual species may persist over decades in the Atacama Desert. Another explanation for these divergent results might be that the conclusions of Arroyo et al. [11] are based on transects spanning a broader altitudinal range than our study.

### Floristic relationships and the barrier function of the absolute desert

Our similarity analysis provides strong evidence for floristic isolation of the North Chilean Andean Desert and North Chilean Coastal Desert clusters (Fig 7). Comparison of floristic dissimilarity values to geographical distances supports these findings (Fig 9). High dissimilarity values between Andean and coastal localities in northern Chile, separated only by short distances (< 200 km), contrasts with lower dissimilarity values between equally distant localities within each cluster (Figs 1 and 9). Several authors [18, 22, 54, 55] have pointed out that floristic isolation appears to be the result of a permanent phytogeographical barrier. The hyperaridity of the Atacama Desert between 18° and 25° S hinders plant exchanges especially from east to west. The divergent floristic composition may, however, also be due to the Andean desert and coastal desert in northern Chile constituting climatically divergent habitats: Coastal plants, for instance, depend on winter rainfall and moisture in form of fog or drizzle, whereas in the Andean desert the development of vegetation is mainly shaped by summer rainfall [2]. Other factors, such as differences in temperature [2] or solar radiation [56] likely also play a role. The two explanations–isolation and ecological divergence–are likely not mutually exclusive and probably represent complementary causes for the floristic divergence of the coastal and Andean deserts in northern Chile. Dispersal across the desert core could take place as downslope dispersal of propagules from the Andes to the coast by landslides, alluvia or even streamflow [57] or from the coast toward the Andes by onshore winds [58, 59], or via dispersal in either direction by animals crossing the desert [60, 61]. Conversely, the scattered, halophilic, extrazonal groundwater oases, e.g., in the Pampa de Tamarugal and on the Rio Loa, cannot have served as corridors or stepping stones since the respective species are not present in either the coastal or the Andean desert vegetation [2, 3]. Only 14 species out of a total of 281 species (5%) are found in both the coastal and Andean localities in northern Chile. This agrees with earlier estimates [18, 22]. Occurrence of species in both coastal and Andean deserts, despite the suggested phytogeographic east-west barrier, could be explained by migration routes through more humid zones connecting coast and Andes located north or south of the absolute desert. Indeed, inspection of geographical distributions of species shared between Andean and coastal localities in northern Chile reveals ranges beyond the latitudinal extension of the absolute desert for eleven species (S5 Table). Based on current distribution data, only three of the 14 species present in coastal and Andean deserts, are likely to have crossed the absolute desert: *Jarava annua*, *Solanum chilense* (Dunal) Reiche and *Polyachyrus sphaerocephalus* D. Don. Climatic conditions in the Atacama Desert have oscillated at least since the last glacial maximum, with more humid periods alternating with dryer periods [62], which have influenced the geographic extent of the absolute Desert, so potentially contributing to the current distribution of plant species across the Atacama Desert. A detailed analysis of these aspects lies beyond the scope of this paper but certainly merits future studies.

Floristic affinities between the north Chilean Andean Desert and the Peruvian Desert cluster, but excluding the north Chilean Coastal Desert (Figs 7 and 8) reinforce the idea of a floristic break along the coast between northern Chile and southern Peru [18, 21, 22]. Our results also lend support to the idea of a north-south corridor for plant exchange along the Andes [24]. Luebert [63] suggested, on the basis of climate studies [5, 7], that generally wetter (summer rain) conditions along the western Andean slopes are likely to maintain such a corridor. About 7% of the flora, accounting for 39 out of 544 species, are shared between localities from the Peruvian Desert and North Chilean Andean Desert. Most of these species exhibit broad distribution ranges, exceeding the Peruvian and North Chilean deserts subject of this study, suggesting that a biotic corridor along the Andean desert is not the only possibility to explain this pattern. Some species, however, range into the Peruvian and northern Chilean deserts (e.g., *Ambrosia artemisioides*, *Exodeconus pusillus* (Bitter) Axelius) (S6 Table), underscoring floristic connectivity between the Andean deserts of Chile and Peru [23, 24]. Unfortunately, only few phylogenetic and population genetic studies investigating this idea are available for Chilean-Peruvian desert plant groups [25, 64–67] and overall distribution ranges for many taxa are very incompletely known.

The analyzed data were compiled from over a dozen different floristic inventories carried out between 1928 and 2017. Thus, a degree of inconsistency within the dataset, due to potential interannual variation in species composition [51], needs to be acknowledged. A possible further source for incongruence of floristic inventories is indicated by Jansen and Dengler [68] who point towards the inconsistent use of plant names based on changing taxonomic concepts and the individual taxonomic understanding of the researcher. Moreover, limited available vegetation data along the northern Chilean and Peruvian Andes highlights the need of further field floristic and vegetation studies. Only two Andean localities from southern Peru could be considered in the analyses. Still, our findings based on floristic data clearly indicate a phytogeographic connection between southern Peruvian and northern Chilean Andes for the first time.

## Supporting information

S1 Table Altitudinal distribution range of species within the four studied transects. indicating lowest (min) and highest (max) elevation of each species along each transect. For Quebrada Aroma elevations below 2400 m refer to actual Quebrada vegetation and data was not included in the analysis.
(XLSX)

S2 Table Literature used for the identification of specimens recorded along the four studied transects. (RIS)

S3 Table Working table including presence/ absence data of 615 species for 21 localities in northern Chile and southern Peru. (XLSX)

S4 Table Working table including altitudinal vegetation and floristic data assessed along the four studied transects. The data was used for the analysis of altitudinal zonation of desert vegetation in the Andean range. Values labelled with ‘-‘ indicate species recorded outside the actual studied transects.
(XLSX)

S5 Table Distribution ranges of species found in the North Chilean Andean Desert and North Chilean Coastal Desert cluster. From 281 species, 14 are found in both clusters accounting for ca. 5% of shared flora.
(XLSX)

S6 Table Distribution ranges of species found in the Peruvian Desert and in the North Chilean Andean Desert cluster. From 544 species, 39 are found in both clusters accounting for ca. 7% of shares flora.
(XLSX)
